# Maize Endophytic Plant Growth-Promoting Bacteria *Peribacillus simplex* Can Alleviate Plant Saline and Alkaline Stress

**DOI:** 10.3390/ijms252010870

**Published:** 2024-10-10

**Authors:** Guoliang Li, Miaoxin Shi, Wenhao Wan, Zongying Wang, Shangwei Ji, Fengshan Yang, Shumei Jin, Jianguo Zhang

**Affiliations:** 1 Heilongjiang Academy of Agricultural Sciences, Harbin 150086, China; 2 Key Laboratory of Saline-Alkali Vegetation Ecology Restoration, Ministry of Education, College of Life Sciences, Northeast Forestry University, Harbin 150040, China; 3 Engineering Research Center of Agricultural Microbiology Technology, Ministry of Education, Heilongjiang University, Harbin 150080, China; 4 Heilongjiang Provincial Key Laboratory of Ecological Restoration and Resource Utilization for Cold Region, Heilongjiang University, Harbin 150080, China; 5 Key Laboratory of Molecular Biology, College of Heilongjiang Province, College of Life Sciences, Heilongjiang University, Harbin 150080, China

**Keywords:** endophytic bacteria, maize, *Peribacillus simplex*, plant growth promote, saline and alkaline stress

## Abstract

Soil salinization is currently one of the main abiotic stresses that restrict plant growth. Plant endophytic bacteria can alleviate abiotic stress. The aim of the current study was to isolate, characterize, and assess the plant growth-promoting and saline and alkaline stress-alleviating traits of *Peribacillus simplex M1* (*P. simplex M1*) isolates from maize. One endophytic bacterial isolate, named *P. simplex M1*, was selected from the roots of maize grown in saline–alkali soil. The *P. simplex M1* genome sequence analysis of the bacteria with a length of 5.8 Mbp includes about 700 genes that promote growth and 16 antioxidant activity genes that alleviate saline and alkaline stress. *P. simplex M1* can grow below 400 mM NaHCO_3_ on the LB culture medium; The isolate displayed multiple plant growth-stimulating features, such as nitrogen fixation, produced indole-3-acetic acid (IAA), and siderophore production. This isolate had a positive effect on the resistance to salt of maize in addition to the growth. *P. simplex M1* significantly promoted seed germination by enhancing seed vigor in maize whether under normal growth or NaHCO_3_ stress conditions. The seeds with NaHCO_3_ treatment exhibited higher reactive oxygen species (ROS) levels than the maize in *P. simplex M1* inoculant on maize. *P. simplex M1* can colonize the roots of maize. The *P. simplex M1* inoculant plant increased chlorophyll in leaves, stimulated root and leaf growth, increased the number of lateral roots and root dry weight, increased the length and width of the blades, and dry weight of the blades. The application of inoculants can significantly reduce the content of malondialdehyde (MDA) and increase the activity of plant antioxidant enzymes (Catalase (CAT), Superoxide Dismutase (SOD), and Peroxidase (POD)), which may thereby improve maize resistance to saline and alkaline stress. Conclusion: *P. simplex M1* isolate belongs to plant growth-promoting bacteria by having high nitrogen concentration, indoleacetic acid (IAA), and siderophore, and reducing the content of ROS through the antioxidant system to alleviate salt alkali stress. This study presents the potential application of *P. simplex M1* as a biological inoculant to promote plant growth and mitigate the saline and alkaline effects of maize and other crops.

## 1. Introduction

There are data indicating that over 800 million hectares of arable land worldwide are affected by salinization, and in China, the area of saline–alkali land accounts for 25% of the total farmland area [1]; sodic soils are characterized by highly alkaline pH (i.e., >pH 8) with a high level of exchangeable sodium [2]. By 2050, it is expected that 50% of the agricultural land will be lost due to soil salinization [3]. How to utilize saline–alkali land and improve the ability of plants to grow on saline–alkali land is an urgent problem to be solved.

In 2018, China’s maize production ranked second only to the United States [4,5]. Maize is not only the main grain crop, but also an important industrial sector, and ensuring the sustained high yields of maize as raw materials is beneficial for maintaining China’s food security [6]. Maize is a moderately salt-sensitive crop, so its growth and development status can be severely affected by salt stress [7]. Maize is affected by salt stress, which can result in a significant decrease in the photosynthetic rate, the growth rate, and dry matter accumulation, and the decline leads to a significant slowdown in maize growth and development [8]. Therefore, increasing the growth and development of maize under saline and alkaline stress is crucially important and meaningful.

Plant growth promotion (PGP) represents a promising solution to strengthen agricultural productivity. Plant-growth-promoting endophytic bacteria (PGPB) and plant-growth-promoting rhizobacteria (PGPR) are essential parts of the microbiome. They are expected to provide new solutions to improve the salt tolerance characteristics of plants [9,10]. PGPR can establish mutualistic relationships with plants through endophytic bacteria, thereby promoting plant growth and enhancing plant resistance to biotic/abiotic stress [11]. PGPR can absorb nutrients such as nitrogen, phosphorus, and iron from the soil and transport them to the rhizosphere for plant absorption to promote plant growth and development [12]. PGPB can alleviate both biotic and abiotic stress on plants by regulating and controlling the photosynthesis and hormone concentration of symbiotic plants, thereby improving plant stress resistance [13,14]. In some cases, microbiome growth stimulation can notably reach levels similar to chemical fertilizers, making the bacterium a sustainable alternative to potentially harmful chemicals in food production [15,16]. Therefore, in the past few decades, products based on PGPB and PGPR have been used in agriculture, but current research on the mechanism of improving plant growth and stress resistance is still very limited.

A number of studies have highlighted *P. simplex*’s potential to act as a PGP microorganism; *Bacillus simplex* was named by Meyer and Gottheil in 1901 and amended by Priest et al. in 1989 [17,18]. *Peribacillus simplex* was the type species of this genus [19], the isolates matching with *B. simplex*, which is currently named *Peribacillus simplex* [20]. *P. simplex* demonstrates a broad range of activity, stimulating growth in a large variety of commercially relevant crops; *P. simplex* isolates stimulate primary root growth and lateral root development, increased shoot and root weight in plants [20,21,22], increased IAA production and high phosphate solubilization [23], increased soil nutrient concentrations (phosphate, magnesium, manganese, and sulfur), and the grain yield and straw weight [24]. Plants inoculated with *P. simplex* showed a change in root architecture due to the emergence of more lateral roots [25]; the *P. simplex* PHYB1 had the highest impact in the highest seed production of *Black cumin* [26]. *P.simplex* isolates enhanced germination, root growth, a high phosphate, siderophore, and zinc solubilization index in wheat [27]; *Peribacillus simplex* had a positive effect on the growth of cultivated rice, and the contents of total nitrogen, total phosphorus, IAA, and chlorophyll in plant leaves were increased [22]. Increased shoot and root weight, IAA production, and high phosphate solubilization were detected in tomato plants treated with *P. simplex* [28]. Bacillus simplex significantly improved photosynthesis yield, potato transpiration rate, water use efficiency, iron uptake, and overall yield [29].

Previous reports suggest that *Bacillus* spp. can increase maize growth and confer salt stress amelioration, and the *P. Simplex* R180 strain coming from wheat increased maize growth significantly compared to the controls [30]. *Bacillus simplex* strain SYM00260 is a recently developed active ingredient for the growth promotion of maize in Brazil. *Bacillus simplex* has sometimes achieved over a quarter of crop yield increase [31,32]. *Bacillus* spp. (*B. safensis*) mitigates NaCl stress and promotes maize growth by modulating ethylene metabolism [31,32]. *P. Simplex M1* was selected from maize roots and its role as an endophytic bacterium in promoting plant growth and alleviating salt alkali stress is worth studying.

With the aim of searching for sustainable plant supplements or alternatives to chemical fertilizers, the use of PGPB has shown great potential, minimizing environmental impacts; thus, the present study was conducted to isolate endophytic isolates of *P. simplex M1* from the maize grown in an alkaline-stressed environment and characterize it for its potential as an endophyte-based bioformulation for enhancing plant growth and alleviating alkaline stress by inoculating maize under salt alkaline stress conditions.

## 2. Results

### 2.1. Sequencing of the 16s rRNA Gene

**Figure 1:**
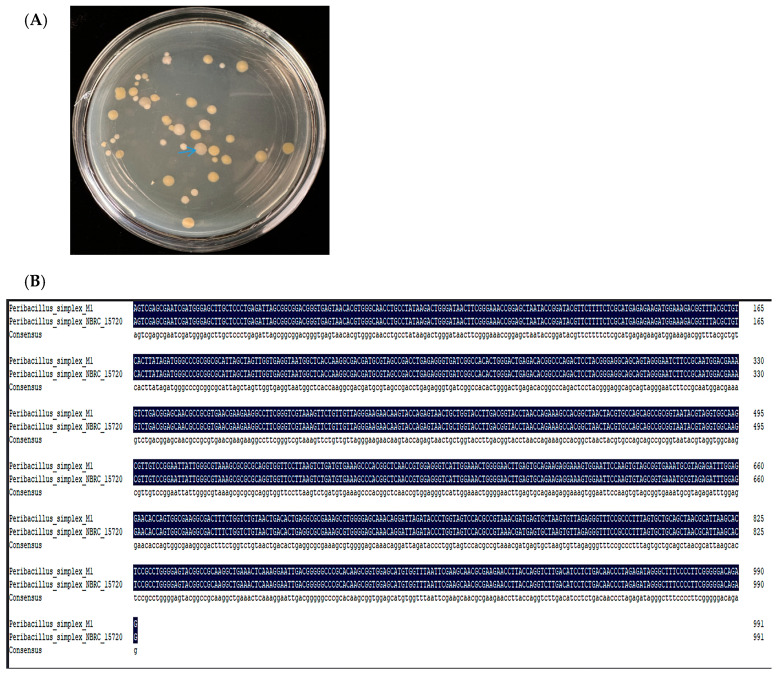
Colonies grown in the NA medium added with 400 mM NaHCO_3_ and blasted with standard bacterial strain in the BERGEY’S MANUAL. (**A**) Colonies grown in the NA medium added with 400 mM NaHCO_3_ (**B**). Blast with standard bacterial strain in BERGEY’S MANUAL.

A bacterial isolate was obtained from the NA (Nutrient Agar) solid medium added with 400 mM NaHCO_3_ (the colony indicated by the arrow in Figure 1A); the colony that can grow on 400 mM NaHCO_3_ medium indicates that the colony has strong salt alkali resistance. However, whether the colonization of this strain into plants can improve their growth and stress resistance is a problem that needs to be addressed in this study. The 16S rRNA gene was amplified by PCR. The amplified 16S rRNA gene DNA PCR product sequence was shown in NCBI (GenBank accession number: PQ358470); Blast the 16S rRNA sequence using the NCBI database. Even though it is just 99.32% identical to the *P. simplex* type strain, most of the Blast result belongs to the genus of *P. simplex*. The analysis revealed that the bacterial isolate corresponds to *Peribacillus simplex* NBRC 15,720 (NR_112726.1) (standard bacterial strain in the BERGEY’S MANUAL) with 100% identity (Figure 1B).

### 2.2. The Bacterium Genome Sequence Analysis

Average nucleic acid similarity (ANI) is one of the most powerful measurement indicators for identifying the genetic relationships of bacterial genomes. The average value of the comparison of all the orthologous protein sequences based on genome quality inspection reflects the evolutionary distance of genome quality inspection. This bacterium genome (GenBank accession number: SUB14754556) has an ANI value of 95.7661 compared to the reference genome (NR_112726.1, *Peribacillus simplex* NBRC 15,720 = DSM 1321); when ANI >95%, it indicates that the two genomes belong to the same species.

Glimmer 3 is used for genome coding gene prediction in this project [33]. Glimmer is the most widely used prokaryotic gene structure prediction software, with high accuracy in prediction results. The analysis of the genome sequencing results of the bacteria showed that a total of 9,153,766 read fragments were obtained, with a length of 5.8 Mbp and a GC content of 34.91%. A total of 5882 coding genes and 123 non coding genes were predicted. Search for the genes related to promoting plant growth and stress tolerance is shown in Table 1.

### 2.3. The Bacterium Trait

**Figure 2:**
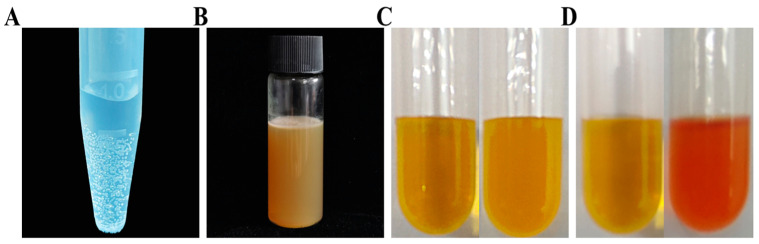
Physiological and biochemical reactions of bacteria. (**A**) Catalase characteristic experiment for catalase positive or negative. (**B**) hydrogen sulfide test for H_2_S production. (**C**) Voges–Proskauer (VP) test for producing acid reaction. (**D**) methyl red test for producing acid reaction.

The morphology of the bacteria is rods and straight, cream-colored, and glossy, with irregular margins. The bubbles appear within half a minute in the catalase characteristic experiment, and it is considered catalase positive (Figure 2A). There were no black precipitates of lead sulfide forming in the hydrogen sulfide test, showing that the bacteria were negative for the hydrogen sulfide test (Figure 2B). We generated a organic compound in the Voges–Proskauer (VP) test (Figure 2C) and produced red acid (Figure 2D) in the methyl red test, showing the ability of the bacteria to produce acidic products using glucose. These features conform to the basic physiological characteristics of *Peribacillus simplex* in the Berger Handbook. Combining 16s RNA, genome sequencing, and physiological index analysis, it is highly likely that this bacterium is *Peribacillus simplex*; this bacterium comes from maize and it is the first time we found this bacterium, so we named this bacterium *P. simplex M1*.

**Figure 3:**
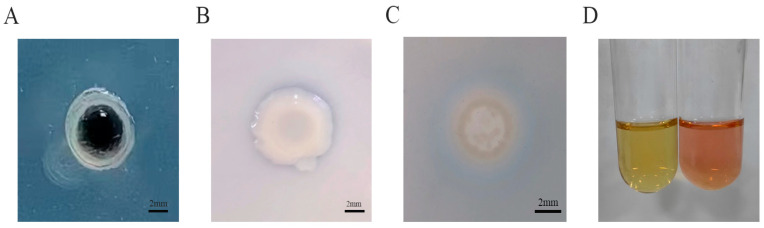
Schematic illustration showing the various tests performed on plates (Petri dishes 9 cm in diameter) for the assessment of the PGP traits of *P. simplex M1*. (**A**) Nitrogen fixation ability on the Nfb culture medium; (**B**) phosphorus solubilization ability on the NBRIP culture medium; (**C**) Siderophore production on the CAS culture medium; (**D**) produce IAA grown on no added tryptophan.

The *P. simplex M1* bacterial isolate can grow normally in an Ashby nitrogen-free culture medium, and this indicates that *P. simplex M1* has nitrogen fixation ability (Figure 3A). A transparent circle around the *P. simplex M1* colony on a NBRIP phosphorus solubilized culture medium (Figure 3B) and a blue to pink color change in the agar under and around a bacterial colony within 30 min of flooding with CAS solution was an indication of siderophore production by the bacterium (Figure 3C), indicating the *P. simplex M1* isolate has the ability to solubilize inorganic phosphate and produce iron carriers. The *P. simplex M1* isolate exhibited the capacity to produce IAA when grown on no added tryptophan (Figure 3D).

### 2.4. Growth of P. simplex M1 under NaHCO_3_ Stress and Different pH

#### 2.4.1. Growth of *P. simplex* M1 under Different Concentrations of NaHCO_3_

**Figure 4:**
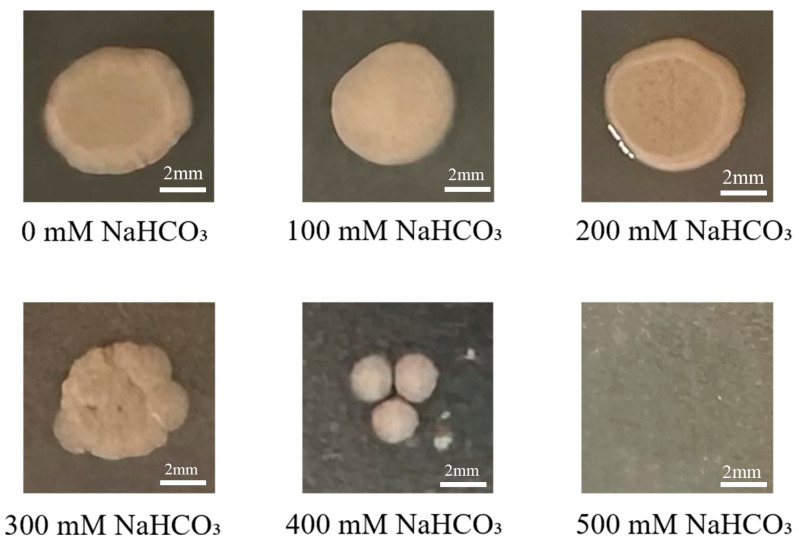
Growth of *P. simplex M1* under different concentrations of NaHCO_3_ stress. A total of 5 μL *P. simplex M1* (OD_600_ = 0.5) were spotted on solid LB media supplemented with the indicated stresses and grew at 30 °C for 3 d. No treatment is a control (CK).

The *P. simplex M1* isolates were able to grow at 0–400 mM NaHCO_3_. When the concentration was increased to 400 mM NaHCO_3_, which showed reduced growth, the bacteria isolate only had little growth, and the bacterial isolate could not grow at 500 mM NaHCO_3_ (Figure 4).

#### 2.4.2. Growth of *P. simplex M1* under Different pH Value

**Figure 5:**
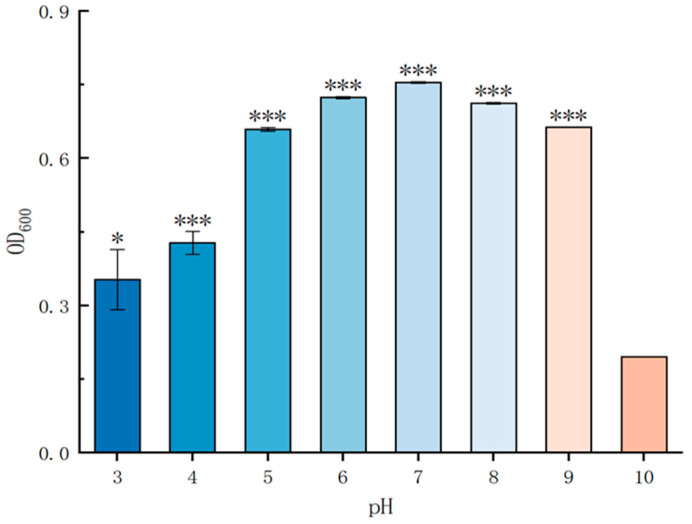
Growth of *P. simplex M1* under different pH conditions. A total of 200 μL *P. simplex M1* (OD_600_ = 0.5) liquid was added to 1 mL sterilized LB liquid medium with different pH values (3–10) at 30 °C with constant shaking at 1300 rpm. The OD_600_ value was measured using an ELISA reader every hour and measured continuously for 24 h. * *p* < 0.05, *** *p* < 0.001, standard error of three biological replicates.

The *P. simplex M1* bacterium can grow at the pH values of 5–9 and be inhibited in growth at pH 3, 4, and 10 in 24 h (Figure 5).

### 2.5. Effect of P. simplex M1 Bacterial Isolates on Growth of Maize

#### 2.5.1. Germination of Maize Seeds under Normal Growth or NaHCO_3_ Stresses Conditions

**Figure 6:**
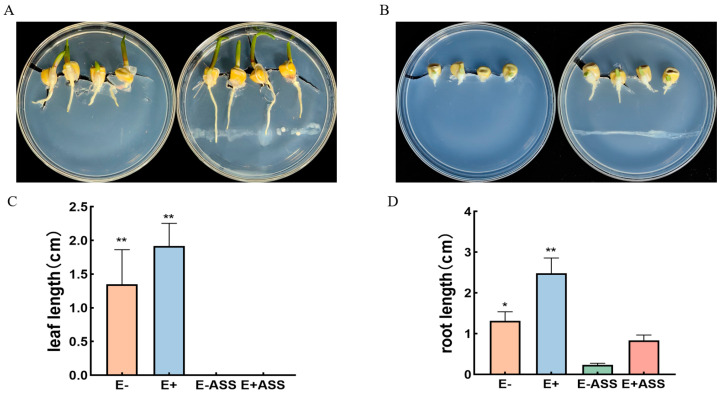
The effect of *P. simplex M1* on maize seed germination. (**A**) Left: the sterile maize seeds were planted on the 1/2 MS; right: the sterile maize seeds in the inoculation with *P. simplex M1* were planted on the 1/2 MS; (**B**) left: the sterile maize seeds were planted on the 1/2 MS + 10 mM NaHCO_3_; right: the sterile maize seeds in the inoculation with *P. simplex M1* were planted on the 1/2 MS + 10 mM NaHCO_3_; (**C**) the leaf length of maize seed germination; (**D**) the root length of maize seed germination for 10 d. The germination percentage of the maize seeds was recorded during 10 d of NaHCO_3_ treatment. Data show the means ± SE of three replicates. At least 50 seeds in each treatment were measured in each repeat. * *p* < 0.05, ** *p* < 0.01, standard error of three biological replicates.

The maize seed germination of the bacterial inoculation group with *P. simplex M1* (germination in 3 days, seed germination rate is 100%) on the culture medium was faster than that in the control group (germination in 4 days, seed germination rate is 100%). Under the stress treatment of 10 mM NaHCO_3_, the germination of maize seeds was inhibited, but the germination rate of the maize seeds in the inoculation group (germination in 5 days, seed germination rate is 100%) was faster than that in the control group (germination in 6 days, seed germination rate is 80%), and the roots and buds also grew longer than the control. So, the maize seeds in the inoculation group had longer roots and shoots than those in the control group in the same period of time, revealing that the bacteria exerted a significant positive influence on early plant growth, namely germination (Figure 6).

#### 2.5.2. TTC, DAB, and NBT Staining

**Figure 7:**
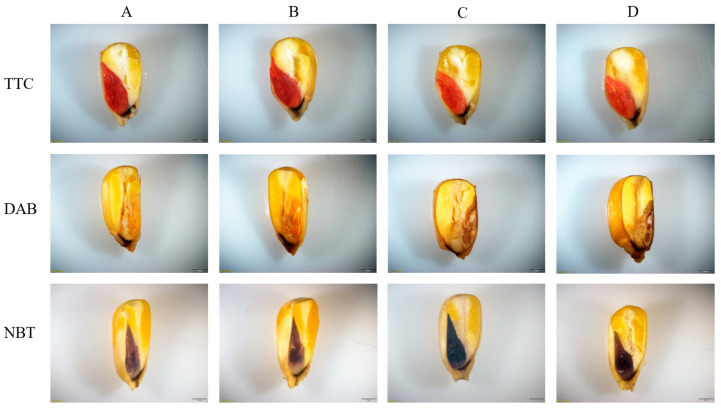
TTC, DAB, and NBT staining of maize seed. (**A**) Each group represents the sterile maize seeds under free salt stress (control); (**B**) the sterile maize seeds in the inoculation with *P. simplex M1* under free salt stress; (**C**) the sterile maize seeds under the 10 mM NaHCO_3_ stress; (**D**) the sterile maize seeds in the inoculation with *P. simplex M1* under the 10 mM NaHCO_3_.

For maize seed tissues, the staining results show that living tissues are stained to varying degrees of red, and the maize seed embryos showed stronger TTC (2, 3, 5-triphenyltetrazolium chloride) staining (deep red) than those seed embryos that were treated with 10 mM NaHCO_3_ (light red), indicating that the seed vitality is affected under the stress. The maize seeds in the inoculation with *P. simplex M1* were stained darker than the control, indicating that *P. simplex M1* is beneficial for increasing seed vitality (Figure 7).

The maize seed was observed by DAB (diaminobenzidine) staining (brown pigment) and NBT (nitro blue tetrazolium) staining (blue). The maize seed embryos showed more dark brown or dark blue staining than those seed embryos that were treated with 10 mM NaHCO_3_; those infected with *P. simplex M1* were lighter in color than those without infection under stress. These results demonstrated that NaHCO_3_ treatment significantly increased ROS levels during seed germination and *P. simplex M1* can decrease ROS content (Figure 7).

The top group is the TTC staining of maize seed vigor; the seed embryos were stained deep red under free salt stress and light red under the 10 mM NaHCO_3_ stress, but those infected with *P. simplex M1* were darker in color than those without infection under stress. The middle group is the DAB staining of maize dynamic accumulation of H_2_O_2_; the seed embryos were stained light brown under free salt stress and darker brown under the 10 mM NaHCO_3_ stress, but those infected with *P. simplex M1* were lighter in color than those without infection under stress. The bottom group is the NBT staining of maize dynamic accumulation of O_2_^−^; the seed embryos were stained light blue under free salt stress and darker blue under the 10 mM NaHCO_3_ stress, but those infected with *P. simplex M1* were lighter in color than those without infection under stress.

### 2.6. The Effect of P. simplex M1 on the Maize Seedlings

#### 2.6.1. *P. simplex M1* Bacterial Strains to Colonize Seedling Roots

**Figure 8:**
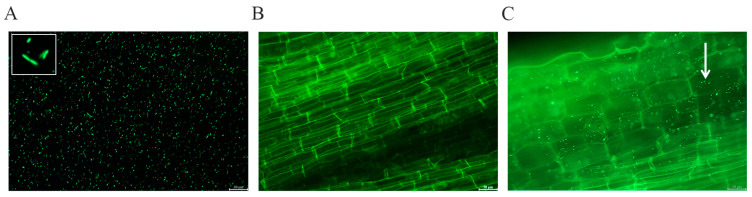
Colonization of *P. simplex M1* on maize seedling root. (**A**) The morphology of GFP-expressing *P. simplex M1*; (**B**) the maize root as a control; (**C**) the colonization of root with GFP- expressing *P. simplex M1*. The white arrow points to a bacterial cluster, scale Bar = 50 µm.

To assess the ability of the *P. simplex M1* bacterial strains to colonize seedling roots we labeled them with GFP following inoculation. The colonization roots with GFP-expressing *P. simplex M 1* bacterial strains were observed using microscopy (Figure 8).

#### 2.6.2. The Effect of *P. simplex M1* on the Maize Seedling

To characterize the effect of *P. simplex M1* on the maize seedling, the *P. simplex M1* strain promoted plant growth in the absence as well as in the presence of salt stress. Both uninoculated and inoculated plants were irrigated with 50 mL water and 50 mL saline solution of 400 mM NaHCO_3_, respectively. The phenotypic growth parameters of the plants recorded in the pot study are presented in Figure 8. The growth of the plants inoculated with *P. simplex M1* was higher compared to the non-inoculated plants under free salt treatments or salt (400 mM NaHCO_3_). The magnitude of leaf SPAD (Soil–Plant Analyzer Development) is a relative chlorophyll content reading that reflects the relative content of chlorophyll. We examined the chlorophyll content (38.4, 33.7, 28.6, and 36.5); the healthier the crop, the higher the SAPD index. The plants’ inoculation with *P. simplex M1* significantly increased the chlorophyll content index of leaves, and this showed that *P. simplex M1* can promote plant growth.

**Figure 9:**
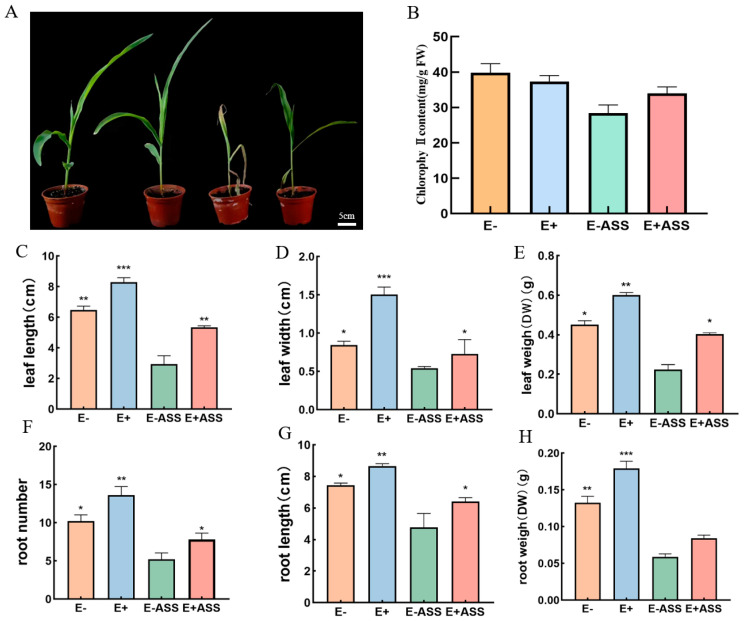
Effect of *P. simplex M1* strain on maize growth parameters after 7 days of cultivation under saline conditions. (**A**) The growth of maize seedling, from leaf to right: uninoculated maize seedling growth under free salt, inoculated maize seedling with *P. simplex M1,* uninoculated maize seedling growth under NaHCO_3_ stress, and inoculated maize seedling with *P. simplex M1* under NaHCO_3_ stress; (**B**) chlorophyll content index; (**C**) leaf length; (**D**) leaf depth; (**E**) leaf dry weight; (**F**) root number; (**G**) root length; (**H**) root dry weight. E- represents uninoculated maize seedlings, E+ represents inoculated maize seedlings, E-ASS represents uninoculated maize seedlings irrigated with 400 mM NaHCO_3_, and E + ASS represents inoculated maize seedlings irrigated with 400 mM NaHCO_3_. The values represent the means of replicates (n = 4) ± standard deviations. Asterisks in superscript indicate a significant difference from the control at 95% between treatments. Each data point is the average of five replicates, and error bars represent ± SE. Error bars indicate ± SD. * Significance at *p* < 0.05, ** Significance *p* < 0.01, *** Significance *p* < 0.001.

The positive effects of *P. simplex M1* on the growth of maize seedlings were observed in the root and leave system. The leaf length (6.476, 8.29, 2.341, and 5.27 cm), leaf depth (0.844, 1.504, 0.542, and 0.818), dry weight of the leaf (0.452, 0.601, 0.2245, and 0.4026 g), the root length (7.438, 8.66, 4.792, and 6.416 cm), root number (10.2, 13.6, 5.2, and 7.8), and dry weight of root (0.1326, 0.1793, 0.059, and 0.084 g) of the uninoculated seeding, inoculated seeding, uninoculated seeding treated with 400 mM NaHCO_3_, and inoculated seeding treated with 400 mM NaHCO_3_ were measured, respectively. When compared to the uninoculated maizes, the *P. simplex M1* strain improved the leaf and root development under four irrigation conditions (Figure 9).

#### 2.6.3. Antioxidant Enzyme Determination of Maize Seedlings under 400 mM NaHCO_3_ Stress

**Figure 10:**
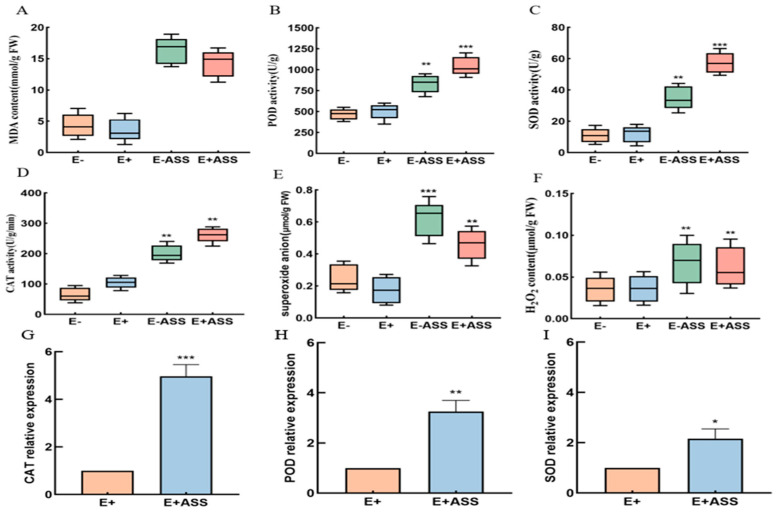
Antioxidant enzyme activity determination in maize seeding. (**A**) MDA content; (**B**) POD activity; (**C**) SOD activity; (**D**) CAT activity; (**E**) superoxide anion; (**F**) H_2_O_2_ content. (**G**) *CAT* relative expression; (**H**) *POD* relative expression; (**I**) *SOD* relative expression. * Significance at *p* < 0.05, ** Significance *p* < 0.01, *** Significance *p* < 0.001.

The MDA (malondialdehyde) content (4.11876, 3.25508, 17.73624, and 14.90944 mmol/g), the CAT (catalase) activity (64.33, 106.12, 192.12, and 247.93 U/g), the POD (peroxidase) activity (450.51, 524.83, 782.21, and 908.58 U/g), the SOD (superoxide dismutase) activity (6.82, 6.70, 43.14, and 61.38 U/g), O_2_^−^ (0.2135, 0.1723, 0.5526, and 0.4143 μmol/g), and H_2_O_2_ (0.026, 0.031, 0.072, and 0.053 μmol/g) was measured in the maize seedlings irrigated with water, maize seedlings irrigated with *P. simplex* bacteria, maize seedlings inoculated with *P. simplex* for 5 days and irrigated by 400 mM NaHCO_3_ stress, and maize seedlings irrigated with *P. simplex* bacteria before NaHCO_3_ stress, respectively (Figure 10). Under salt-free treatment, the activities of the antioxidant enzymes (MDA, CAT, POD, and SOD) of maize irrigated with water and *P. simplex* bacteria were basically the same, but after NaHCO_3_ treatment, they were significantly different. The production of MDA can exacerbate membrane damage. MDA can be used to assess the degree of membrane lipid peroxidation, indirectly determining the extent of membrane system damage and plant stress resistance. The MDA content of maize seedlings irrigated with *P. simplex M1* bacteria is lower than that in the maize seedlings under 400 mM NaHCO_3_ stress. *P. simplex M1* bacteria can reduce stress damage to plant membranes.

The expression of *CAT*, *POD,* and *SOD* genes was increased under the conditions of 400 mM NaHCO_3_, which is nearly 2–5 times higher than free salt. The CAT activity, the POD activity, and the SOD activity of the maize seedlings inoculated with *P. simplex M1* are higher than that of maize seedlings under stress; these important antioxidant enzymes can clear free radicals in the body, thereby protecting cells from oxidative damage, indicating that *P. simplex M1* significantly increases antioxidant activity in maize seedlings and reduce plant damage.

## 3. Discussion

Soil alkalinity has a notable effect on organic matter, nitrogen, carbon, and microbial biomass [34]. The utilization of beneficial microbes to enhance crop productivity under stressed environmental conditions is an eco-friendly approach [35,36]. The microbes produce advantageous effects on plants, such as phosphate solubilization and nitrogen fixation [21,37]. PGPB for not only enhancing plant growth but also mitigating abiotic and biotic stresses is essential [38,39].

A bacterium was isolated from the roots of maize grown in saline–alkali soil with a pH value of 8.5. Through the genomic research of bacterial colonies, a total of 515 nitrate process genes, 19 iron ion-binding, and 60 IAA production genes were predicted in the *P. simplex M1* genome sequence (Table 1). The presence of these genes indicates that this bacterium has the molecular basis for promoting plant growth. Maize requires the highest amount of nitrogen, so the N content in plants is a critical growth macro-element. The *P. simplex M1* bacterial isolate can convert nitrogen that plants cannot use in the environment into nitrogen that plants can absorb. There were 515 nitrogen compound metabolic process genes in the *P. simplex M1* genome (Table 1), such as the nitrogen fixation (*nifS*) genes and Nitrogen control A (*NtcA*) genes which were annotated in the *P. simplex M1* genome. *nifS* are required components of the enzymatic module encoding nitrogenase [40]. *NtcA* transcription factor operates global nitrogen regulation in these photosynthetic organisms [41]. When *P. simplex M1* colonizes the maize, these growth-related genes also enter the maize plant and may be expressed during seed germination and plant growth; these genes help nitrogen utilization and cycling in plants, thereby enhancing plant growth. *P. simplex M1* promote plant growth through processes similar to these molecular mechanisms.

Phosphorus is often one of the limiting factors for high crop yields [22]. A total of 160 genes in the *P. simplex M1* genome are involved in the phosphorus metabolic process; *P. simplex M1* has the ability to dissolve phosphorus (Figure 3), making it easier for plants to absorb and utilize the dissolved phosphorus. Growth stimulation has most commonly been attributed to direct growth promotion via IAA or siderophore secretion [24,42]. *P. simplex M1* capacity to produce IAA and iron carriers (Figure 3C,D) to directly promote plant growth. The bacteria were able to grow normally under 400 mM NaHCO_3_ or pH value from 5 to 9 (Figure 4), indicating that the bacteria can survive and function under saline–alkali conditions.

*P. simplex* has demonstrated a good root colonization potential and persistence in several commercial plants [43]. *P. simplex M1* has the ability to colonize the roots of the maize (Figure 8). *P*. *simplex* has a positive effect on the growth of cultivated plants [20,22,25,27,28,29,30,44]. The maize seed inoculated with *P. simplex M1* showed stronger TTC staining (Figure 7) and germinated faster (Figure 6) than the controls (distilled water). *P. simplex M1* exerted a significant positive influence on early plant growth, namely germination rate. Seed germination was modulated by various factors including epigenetic modifications, hormone transport, and ROS signaling [45]. Saline irrigation negatively affected plant growth compared to the set irrigated by non-saline water (Figure 9). These results demonstrated that *P. simplex M1* significantly increased seed vigor and decreased ROS levels during seed germination.

There are a total of 16 antioxidant activity genes in the genome of bacteria, including catalase genes, superoxide dismutase genes, thiol-disulfide oxidoreductase resA, thioredoxin-dependent thiol peroxidase, and so on. CAT is an H_2_O_2_ scavenger that dissociates H_2_O_2_ into O_2_ and H_2_O [46]. Peroxidase can remove H_2_O_2_ by oxidizing the substrate. The activities of SOD and POD play an important role in scavenging ROS. The infection of *P. simplex* significantly increased the activity of the antioxidant enzymes in maize seedlings, which indicated that the addition of microbial inoculum was helpful for maize to remove excessive ROS.

**Figure 11:**
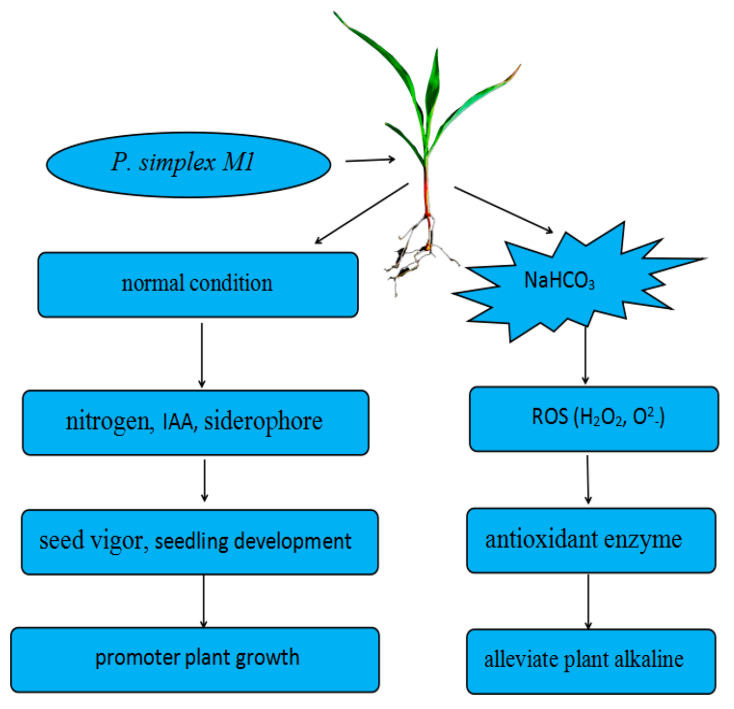
A model for the mechanism underlying *P. simplex M1* promoted maize development. The *P. simplex M1* has the ability to fix atmospheric nitrogen, produce IAA, produce siderophore, and enhance the antioxidant enzyme activity. When the maize is inoculated with *P. simplex M1*, *P. simplex M1* can increase seed vigor and seedling development (root length, leave length, and so on), thereby promoting maize growth. When the maize was treated with NaHCO_3_, with the increase in ROS in plants, the activity of antioxidant enzymes caused by *P. simplex M1* also enhanced, which alleviated the saline–alkaline stress on plants.

A resistant maize *P. simplex M1* strain promotes growth by fixing atmospheric nitrogen, solubilizing phosphorus, producing IAA, producing siderophore, and alleviating saline and alkaline stress by increasing oxidative mechanisms (Figure 11).

## 4. Materials and Methods

### 4.1. Isolation and Identification of Selected Bacterium

The endophytic bacterial strains were selected from the roots of maize (B46, Salt tolerant variety) grown in saline–alkali soil with pH 8.5 on the Songnen Plain of Heilongjiang Province in Northeast China. The maize was dug out from the growing field, the roots were washed with sterile water for 5 min and cleaned with NaClO for 1 min, and then the roots were ground in a sterile centrifuge tube and resuspended in sterile water to obtain a bacterial suspension. A volume of 200 μL of the bacterial supernatant was spread onto NA (Nutrient Agar) solid medium added with 400 mM NaHCO_3_ and inverted in an incubator at 30 °C for 3 days, and after the colonies had grown, they were purified separately on new NA solid medium. The 16S rRNA gene was amplified by PCR for these colonies. The sequences of primers used for amplification were universal primers 27F (5′-AGAGTTTGATCCTGGCTAG-3′) and 1492R (5′-CTACGGCTACCTTGTTACGA-3′), and the amplified DNA PCR products (~1540 bp for 16S rRNA sequences) were commercially sequenced (Kumei Biotechnology Co., LTD, Changchun, China). The sequenced results were compared to those in the GenBank database using the BLAST algorithm (National Center for Biotechnology Information, Bethesda, MD, USA).

### 4.2. The Bacterium Genome Analysis

We sent the selected *P. simplex M1* strain to Magi Gene Biotechnology Company (Guangzhou, China) for whole genome sequencing, and the genomic DNA was extracted by Guangdong Magigene Biotechnology Co., Ltd. (Guangzhou, China) using a DNA extraction kit of the corresponding sample. The ALFA-SEQ DNA Library Prep Kit (was used for qualified DNA samples for library construction. The PE150 sequencing of the constructed amplicon libraries was performed using Illumina or MGI platforms (Guangdong Magigene Biotechnology Co., Ltd., Guangzhou, China). Quality control was carried out on the raw data, and the high-quality sequence obtained was used for downstream data analysis. The tool SPAdes v3.13.0 was used to perform de novo splicing on the clean data after data filtering and quality control to obtain high-quality contig fragments. The prediction of genome components includes the prediction of coding genes, repeat sequences, non coding RNA, and prophage. Genome-wide Blast searches were performed on the following databases: NR (Non-Redundant Protein Database), Swiss-Prot, GO (Gene Ontology), KEGG (Kyoto Encyclopedia of Genes and Genomes), COG (Clusters of Orthologous Groups), etc., to predict gene function.

### 4.3. Evaluation of the Bacterium Traits

Referring to the classification explanation of BERGEY’S MANUAL (Second Edition Volume Three, pp. 115–116), we selected indicators such as catalase characteristics, methyl red test, Voges-Proskauer, and gelatin hydrolysis to detect the physiological and biochemical characteristics of bacteria. The experimental method is as follows: Inoculate the bacterial colonies in a tube and add 2 mL of 3% hydrogen peroxide solution; if bubbles appear within half a minute, it is considered positive. Some bacteria can decompose sulfur-containing amino acids, producing hydrogen sulfide and lead in the culture medium, forming black precipitates of lead sulfide, which are positive for the hydrogen sulfide test. The methyl red test is used to detect the ability of bacteria to resolve glucose and produce organic acids; the culture medium turns red, indicating a positive result in the methyl red test. The Voges-Proskauer test is used to determine the ability of bacteria to produce acidic products using glucose; the bacteria generate a red compound, indicating a positive VP reaction. Escherichia coli does not produce red compounds, indicating a negative reaction. Some bacteria can produce gelatinase. Gelatinase can dissolve gelatin, causing it to lose its coagulation power.

The ability of *P. simplex M1* to fix atmospheric nitrogen on an Ashby nitrogen-free culture medium was tested [47]. Inoculate 2 μL of activated bacterial solution onto a culture medium plate at 30 °C for 24 h; if there is a transparent circle around the colony, it indicates that the bacterium has nitrogen fixation ability. The ability to solubilize inorganic phosphate was tested by inoculating the bacterial isolates in NBRIP (The National Botanical Research Institute’s phosphate) culture medium according to Mehta S [48]. Inoculate 2 μL of activated bacteria onto a NBRIP plate at 30 °C for 24 h. If there is a transparent circle around the colony, it indicates that the bacterium has the ability to dissolve phosphorus. The estimation of IAA was carried out by the method that has been described in Bric et al. [49]. Add 40 mL of the 2.5 mg/mL tryptophan solution into 160 mL DF liquid culture medium. Inoculate the activated bacteria into the above culture medium at 30 °C and 135 rpm for 24 h, take 2 mL of supernatant, and add 50 μL of 50% orthophosphate solution and 4mL of Salkowski reagent. If the supernatant turns red, an IAA enzyme is produced. Siderophore production was detected on CAS (Chromogenic Agar for Spore) culture medium by the method that has been described in Yeole, R et al. [50]. Inoculate 2 μL of the strain on a CAS plate. Incubate at 30 °C for 3 days. The formation of a yellow green halo indicates the production of iron carriers.

### 4.4. Abiotic Stress-Tolerance Estimation for P. simplex M1

To assess the salinity tolerance of the isolated strain, 5 μL *P. simplex M1* (OD_600_ = 0.5) were spotted on solid LB medium plates supplemented with various NaHCO_3_ concentrations (0–500 mM) and incubated for 48 h at 30 °C.

To estimate the maximal growth and minimal inhibitory pH value, *P. simplex M1* was further tested in liquid LB. The bacterial isolates were subjected to evaluate their abiotic stress tolerance including pH (3–10). Add 200 μL *P. simplex M1* (OD_600_ = 0.5) liquid to 10 mL sterilized LB liquid medium added with different pH values (3–10) and mix well to cultivate the bacterial isolates for 24 h at 30 °C with constant shaking at 130 rpm. The OD_600_ value was measured using an enzyme-linked immunosorbent assay (ELISA) reader every hour and measured continuously for 24 h. The experiments were performed in triplicate.

### 4.5. Effects of P. simplex M1 on Maize Plants Using Inoculation Experiments

#### 4.5.1. *P. simplex M1* Affect Maize Seed Germination Assay

The cell pellet of *P. simplex M1* suspension was obtained from a 10 mL fresh overnight culture by 5 min centrifugation at 4000 r/min 10 min. The bacterial pellet was resuspended in 10 mL of distilled sterile water (OD_600_ = 0.8) and vortexed before being used for seed treatment.

Maize seeds (*Zea mays* cv. NC236) were obtained from the Heilongjiang Academy of Agricultural Sciences. NC236 is a salt-sensitive cultivar. To observe the germination of seeds under normal and NaHCO_3_ stress conditions, the seeds were sterilized with 75% alcohol, and 4 maize seeds were planted in sterilized 1/2 MS culture or 1/2 MS culture added with 10 mM NaHCO_3_ medium. The control group did not add *P. simplex M1* solution, while the experimental group needed to use a pipette to aspirate 100 μL of actively growing bacterial suspensions and draw lines on the culture medium of the experimental group. The plates were incubated in a growth chamber at 25 °C under a 16 h light/8 h dark cycle. When the radicle protruded from the seed coat, the seeds were considered germinated. After 10 days, observe the germination of maize seeds and take photos to record.

Maize seeds were sterilized and divided into four groups. The first group seeds were treated with H_2_O for 6 h; the second group seeds were treated with *P. simplex M1* bacterial soaking for 6 h; the third group seeds were treated with H_2_O for 6 h first, followed by 100 mM NaHCO_3_ stress for 30 min; and the fourth group seeds were treated with *P. simplex M1* bacterial soaking for 6 h first, followed by 100 mM NaHCO_3_ stress for 30 min. The maize seeds were immersed in different solutions for testing seed vigor (TTC) or ROS content (DAB and NBT).

The TTC is a lipid-soluble photosensitive complex that can be used to detect the viability of seeds. The detection mechanism is that TTC itself can serve as a redox indicator, and dehydrogenases can reduce TTC in living cells. For seeds or plant tissues, the staining results show that living tissues are stained to varying degrees of red, while dead or lifeless tissues are not stained. The control and treated maize seeds were longitudinally split into two parts, then immersed in 0.5% TTC solution at 30 °C for 2 h in a dark chamber, and then rinsed with double distilled water, and finally photographed [51].

Triplicates of the treatment were maintained. For hydrogen peroxide (H_2_O_2_), DAB staining was used to detect the active site of peroxidase in cells, and was conducted as described previously [52]. The control and treated maize seeds were longitudinally split into two parts, immersed in 1% DAB solution at 30 °C for 12 h, and then photographed.

The NBT staining method is used to detect superoxide anions in plant living tissues. Superoxide anion is an oxygen-containing free radical that can reduce NBT to form insoluble blue formamide compounds. After staining, the areas where superoxide anions aggregate appear blue to dark blue. The maize seeds were immersed in 0.02% NBT solution at room temperature for 15 min in darkness. The histochemical detection of O_2_^−^ was conducted as described previously [52].

#### 4.5.2. *P. simplex M1* Affect Maize Seedlings Using Pot Experiment

To prove that *P. simplex M1* can colonize the maize, the maize seeds (NC236 variety) were sown in pots with soil, and then placed in a growth chamber for cultivation. Relative humidity (RH) varied around 60%, with 25 °C and photoperiod of 14 h light and 10 h dark. The GFP (green fluorescent protein) was used as a test. The construction of GFP-labeled *P. simplex M1* referred to Lifeng Guo [53]. The cell pellet of GFP-labeled *P. simplex M1* suspension (OD_600_ = 0.8) was obtained from a 50 mL fresh overnight culture by 5 min centrifugation at 4000 r/min 10 min. The bacterial pellet was resuspended in 50 mL of distilled sterile water. The maize seedlings (at the four-leaf stage) in the pot were irrigated with the 50 mL GFP-labeled *P. simplex M1* solution for 7 days, and the maize root was visualized by a confocal scanning laser microscope (CSLM) after successful transformation with GFP-labeled *P. simplex M1* solution.

To test *P. simplex M1*’s effect on maize seedlings, the cell pellet of *P. simplex M1* suspension (OD_600_ = 0.8) was obtained from a 50 mL fresh overnight culture by 5 min centrifugation at 4000 r/min 10 min. The bacterial pellet was resuspended in 50 mL of distilled sterile water. The maize seedlings (at the four-leaf stage) with similar growth and development levels were watered with four irrigation treatments. The first group of plants was irrigated into the pots only with 50 mL water (non-saline, as the control); the second group of plants was irrigated with the 50 mL resuspended *P. simplex M1* solution; the third group of plants was irrigated with 50 mL water for 5 days (maintain consistency with the fourth salt treatment time), then the pot was irrigated with the 50 mL saline solution of 400 mM NaHCO_3_; and the fourth group of plants was inoculated with 50 mL *P. simplex M1* solution for 5 days, then irrigated with the 50 mL saline solution of 400 mM NaHCO_3_.

A total of 20 days after four irrigation treatments, the leaf chlorophyll content index was recorded using a chlorophyll content meter (Hansatech instruments, Model CL-01). Leave and root lengths were assessed using a ruler, and the dry weights (DW) of the roots and leaves were measured after drying in an oven at 70 °C for 72 h. Student’s *t*-test was conducted to test for significant differences. In order to evaluate enzymatic antioxidants, the activity of SOD [54], CAT [55], and POD [55] was determined using the methods described earlier, respectively.

In order to observe the differential antioxidant gene expression in the presence and absence of stress, RNA was extracted using the RNeasy Plant Mini Kit (Qiagen, Hilden, Germany) from the leaves of maize which were treated with four different methods. The full-length cDNA was obtained using the Takara reverse transcription kit (Takara, Tokyo, Japan), and the expression of *CAT* (the primer sequence *CAT* qPCRF: AGCGTTCTCTCCAAGTGCAA and *CAT* qPCRR:CGCAGTAAGCTGGGTCTTCA), *SOD* (the primer sequence *SOD* qPCR-F:CGGTGAGCCTAATGGAGACG, *SOD* qPCRR:ACTCTGGGCGCCTATTTTGA), and *POD* genes (the primer sequence *POD* qPCRF:TAAAGGCAACCCGATGACCC, *POD*PCRR:AGATCCGCGCTAATGGTCAG) in the *P. simplex M1* genome was analyzed by qRT-PCR (reverse transcription-quantitative polymerase chain reaction). As an internal reference, the expression of the *Maize18S* gene (the primer sequences were Maize18SF:5’-CAACCATAAACGATGCCGA-3’ and Maize18SR: 5’-AGCCTTGCGACCATACTCC-3) was examined. RT-qPCR was carried out in IQ5 real-time PCR equipment (Bio-Rad, Hercules, CA, USA) with SYBR green (Takara, Tokyo, Japan).

### 4.6. Statistical Analysis

All the data in the manuscript were analyzed by *t*-tests using the SPSS 22.0 software (SPSS Inc., Chicago, IL, USA). For appropriate statistical power, all the assays were repeated three times and the values are presented as means standard errors. Differences were considered significant when the *p* value was less than or equal to 0.05.

## Figures and Tables

**Table 1:** The genes related to promoting plant growth and stress tolerance.

Gene Function	Gene Number	Genes Name
nitrogen compound metabolic process	515	*ntcA* and *nifS*
phosphorus metabolic process	160	*Pqq*, *pstA*, *pstB*, and *pstS*
IAA production	60	*AdhR*, *aroA*, and *trpA*
Iron carrier synthesis	19	*Fer*, *pksS*, and *rub3*
antioxidant activity	16	*CAT*, *SOD*, and *peroxidase*

## Data Availability

All the data supporting the findings of this article can be found in the paper.
